# Green solution to riparian pollution: *Populus alba* L. potential for phytoremediation and bioindication of PTEs along the Sava river

**DOI:** 10.1016/j.heliyon.2024.e28183

**Published:** 2024-03-18

**Authors:** Zorana Miletić, Milica Jonjev, Snežana Jarić, Olga Kostić, Dimitrije Sekulić, Miroslava Mitrović, Pavle Pavlović

**Affiliations:** Department of Ecology, Institute for Biological Research ‘Siniša Stanković’, University of Belgrade, Bulevar despota Stefana 142, Belgrade, Serbia

**Keywords:** *Populus alba*, Sava river, Potentially toxic elements, PTE accumulation, Phytoremediation, Bioindication

## Abstract

This study addresses the potential of *Populus alba* L. for bioindication and phytoremediation of the contaminated lower part of the Sava River. The main objectives are to assess soil contamination with potentially toxic elements (PTEs: As, B, Cd, Cr, Cu, Li, Ni, Pb, Sr, and Zn), evaluate their availability, and assess the phytoremediation and bioindication potential of *Populus alba*. Quantification of the PTE contents was performed using inductively coupled plasma optical emission spectroscopy (ICP-OES), while bioindication and phytoremediation potential were evaluated using accumulation indices. The study revealed phytotoxic contents of Cr, Cu, Ni and Zn in the riparian soils of the lower Sava River. The percentage of available Cd was high, but due to its low total content, its phytotoxic potential is limited. According the metal accumulation index, *Populus alba* exhibits significant potential to accumulate the PTEs studied (with accumulated toxic contents of B, Cr, Li, Sr, and Zn). The ability of *Populus alba* to accumulate and bioindicate Cd, Cr, and Ni is promising, as is its ability to potentially remediate B, Cd, and Zn. Copper deficiency in leaves resulted in a reduction in photosynthetic performance, but without visible morphological symptoms. The reduced photosynthetic capacity serves as an adaptive strategy for this species in response to toxic levels of PTEs. Since *Populus alba* is widely distributed in European riparian forests, it is a good candidate to address soil contamination through phytoremediation and bioindication techniques.

## Highlights

• Soils in the lower course of the Sava River contain phytotoxic Cr, Cu, Ni, and Zn.
• *Populus alba* has a potential for bioindication of Cd, Cr, and Ni in riparian soils.
• This species also has a great potential of for phytoextraction of Cd, B, and Zn.
• Based on MAI, *Populus alba* has great potential for the accumulation of PTEs.
• *Populus alba*: good candidate for pollution control in Europe's riparian ecosystems.

## 1 Introduction

Pollution of water bodies and coastal areas is gaining increasing attention of researchers. Studies have extensively examined the adverse effects of potentially toxic chemical elements (PTEs), the long-term accumulation of which in river sediments and riparian soils is of concern. PTE contents exceeding environmental standards are detrimental to biodiversity in these ecosystems [1]. Recent focus has been on potentially toxic elements (PTEs), particularly heavy metals (Cd, Cr, Cu, Hg, Ni, Pb, Zn) and arsenic (As), in stream and coastal river sediments. These elements are identified as priorities in the European Commission's Water Resources Management Guidelines [2].

The ecological significance of the Sava River and its riparian zone is evident through numerous designated protected areas spanning four countries: Serbia, Bosnia and Herzegovina, Croatia and Slovenia. Encompassing a 97,713 km^2^ catchment area, the Sava is the Danube's third longest tributary. Across 167 designated areas, including 6 Ramsar sites and 8 national parks, crucial bird and plant habitats are conserved nationally and under the Natura 2000 network [3].

PTE pollution in the Sava River follows a spatial pattern: upper reaches experience mineral weathering, middle reaches show agricultural contribution, and lower reaches have elevated pollutant levels due to industrial processes and untreated wastewater discharge [4]. The Sava River is classified as a moderately polluted river in Europe in terms of pollution by PTEs present in both water and sediment [5]. The recent studies have shown that the lower river channel is the most polluted part of the Sava River [[5], [6], [7]].

The accumulation of PTE in plants reflects their availability in the soil, species-specific properties, and plant's functional characteristics. Soil properties, such as physicochemical attributes, influence the accessibility of elements. Some plants can accumulate PTEs at varying concentrations relative to soil solution content [8]. The uptake and transport of elements by roots and aboveground parts are critical for bioindication of soil contamination, which includes analysis of element content of roots and leaves [9]. Toxicity to plants depends on species, metal classification, soil properties, and other factors. In general, higher pH, clay, and organic matter reduce element availability to plants [10,11].

Plants aid in bioindication and biomonitoring of toxic elements [12]. Woody plants are preferred for biomonitoring offer wide distribution, longevity, ease of sampling, and PTE accumulation [13]. The tolerance and accumulation ability of certain plant species can mitigate pollution and restore degraded coastal ecosystems. The use of native species for phytoremediation is essential because of their ecological adaptations [14]. The identification of PTE-resistant native species remains a focus. Indeed, species from the *Salicaceae* family, especially those from the *Populus* genus, are well-known for their potential in phytoremediation and the revitalization of polluted habitats, but still, their potential is species-specific [15,16].

Sometimes plants can accumulate PTEs when exposed to them in high concentrations. However, these elements can interfere with photosynthesis, suggesting that PTEs are one of the most important stressors for photosynthesis [17]. Photosystem II (PSII) is considered to be the part of the photosynthetic apparatus that is most sensitive to light‐induced damage, and damage to PSII is often the first response to stress in plants [18]. Therefore, in this study, measurements of induced fluorescence will be used to provide insights to a plant's ability to tolerate stress induced by PTEs and to gain insight into the extent to which this stress has affected the photosynthetic apparatus.

The aim of this study is to: (1) evaluate the content of As, B, Cd, Cr, Cu, Li, Ni, Pb, Sr, and Zn in the soils of lower contaminated part of the Sava River, (2) assess the availability of these elements in the soils, (3) determine the soil properties that could influence the uptake of the elements by the plants, (4) evaluate the ability of *Populus alba* L. to accumulate As, B, Cd, Cr, Cu, Li, Ni, Pb, Sr, and Zn in its roots and leaves, (5) determine the potential of this species for bioindication, and/or phytoremediation of these elements in contaminated riparian soils, (6) evaluate the sensitivity of the photosynthetic apparatus to pollution-induced stress by examining the effects of PTEs on leaf photosynthetic efficiency (Fv/Fm).

The strength of this study is that it provides regional information that is crucial for understanding the unique dynamics of PTE uptake in *Populus alba* in riparian soils of the Sava River ecosystem (Western Balkans), while the main limitation of the study is the lack of a clean, PTE-free control site.

## 2 Materials and methods

### 2.1 Study area and sampling

**Fig. 1:**
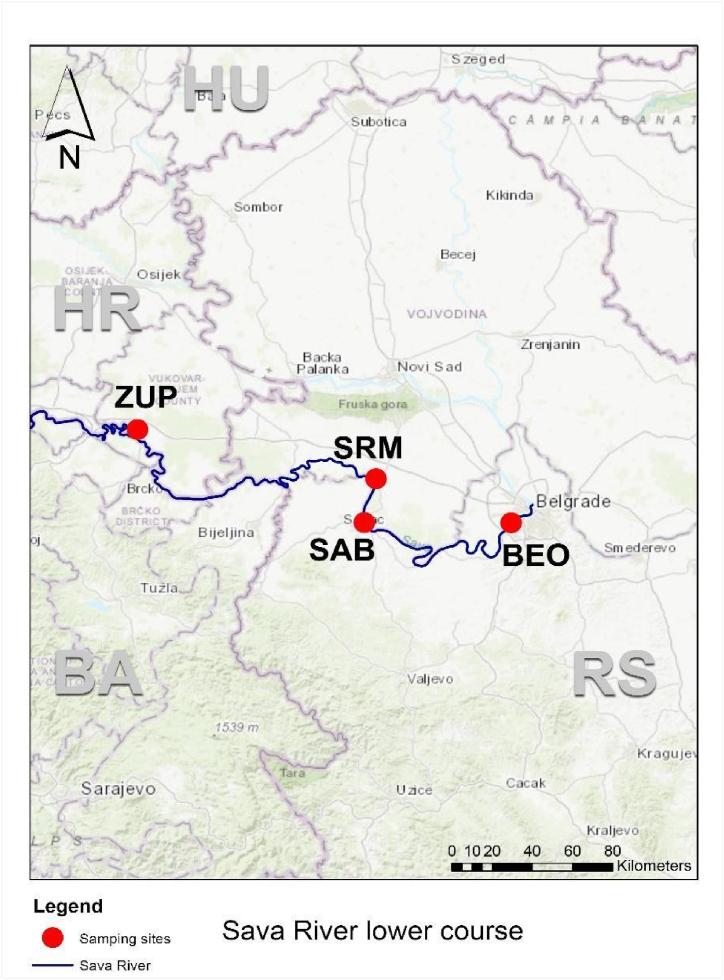
Sampling sites in the Sava River lower course.

To assess the extent of soil contamination in the riparian zone, a comprehensive sampling campaign was conducted during the GLOBAQUA project [19]. Sampling was conducted during September 2015, at locations along the lower reaches of the Sava River, following established sampling protocols that govern the collection of samples and their preparation for subsequent analyzes. Sampling locations included Zupanja (ZUP), Sremska Mitrovica (SRM), Sabac (SAB), and Belgrade (BEO) within the lower segment (Fig. 1). Each of these sampling sites had different forms and extents of anthropogenic pollution, reflecting the different types and intensities of human-induced activities (Table S1).

The soil samples were taken from specific locations, each 10–15 m from the river bank. The choice of location was justified by the fact that this area is prone to flooding during times of high water events. For each site, composite samples were compiled from 5 subsamples obtained from different sampling locations along the riverbank. These subsamples were collected at depths ranging from 0 to 30 cm. After collection, the composite samples were transferred to PVC buckets and mixed thoroughly to ensure uniformity before further processing.

To facilitate analysis, the samples were stored in the dark at a temperature of 4 °C prior to the determination of trace elements. In the laboratory, the samples were air dried and then ground in a stainless steel mill. The resulting material was then sieved through a 2-mm stainless steel sieve and then stored in clean polypropylene bags to prepare it for further analysis.

Representative specimens of the species *Populus alba* were carefully selected at each site, making sure that they were of approximately the same age. Leaves were collected from the stems of three to five selected specimens at a height of 1.5–2 m. Root samples were also collected from the rhizosphere zone of these individuals. These collected samples were then used to create a composite sample for each site.

The root and leaf samples were washed with distilled water and then dried in an oven at 75 °C until they were uniform in weight. After the drying process, the samples were ground using a laboratory mill (Polimik, Kinematica AG). The material was then sieved through a stainless steel sieve with a diameter of 1.5 mm. These processed samples were then prepared for further analysis to determine the content of chemical elements.

### 2.2 Physical and chemical properties of the soil samples

Soil physical properties were evaluated in accordance with the International Soil Texture Triangle [20], which allowed the determination of sand, silt, and clay content (%). Soil pH was determined by measurements in water (H_2_O) using an inoLab 7110 pH meter from WTW (Germany). Soil texture evaluation was based on the Atterberg sedimentation technique combined with a pipette method performed in a solution of 0.4 M tetrasodium diphosphate (Na_4_P_2_O_7_) [21]. To determine the soil organic carbon content (%), a titration technique was used. Here, (NH_4_)_2_Fe(SO_4_)2 × 6H_2_O was used after the samples had been subjected to digestion with a dichromate-sulfuric acid solution. The method for determining the organic carbon content was based on Simakov's modification of the Turin method [22].

### 2.3 PTE content in soil and plant samples

For quantification of total element content, 0.5 g of soil material was subjected to microwave-assisted digestion using the CEM Mars 6 system. Aqua regia, consisting of 3 ml HNO_3_ and 9 ml HCl, was used for the digestion. Subsequently, the concentrations of the potentially toxic elements (As, B, Cd, Cr, Cu, Li, Ni, Pb, Sr, and Zn) were determined using inductively coupled plasma optical emission spectroscopy (ICP-OES) with a Spectro Genesis instrument. To ensure the accuracy of the results, an analysis of a standard reference material (clay soil - ERM-CC141, certified by EC-JRC) was performed. The recoveries from this analysis were in the range of 95–110%. Diethylene triamine pentaacetic acid (DTPA) was used to determine the bioavailable content of PTEs according to the method described by Lindsay and Norvell [23].

For the determination of the total element content in the plant material, a 0.3 g sample was subjected to microwave-assisted digestion using the CEM Mars 6 system. The digestion was performed with a mixture of concentrated nitric acid (HNO_3_, 9 ml) and hydrogen peroxide (H_2_O_2_, 30%, 3 ml). Subsequently, the content of PTEs were quantified using inductively coupled plasma optical emission spectroscopy (ICP-OES) with a Spectro Genesis instrument.

To validate the accuracy of the results, a standard reference material (Beach leaves - BCR-100, certified by EC-JRC) was subjected to analysis. The recoveries derived from this assessment were in the range of 94–107%.

All measurements were performed in 5 replicates to ensure the reliability of the data. Element contents were presented as milligrams per kilogram of dry weight (mg kg^−1^ d.w.) standardized. The detection limits for the studied elements in the soil samples were as follows (in mg kg^−1^): As – 0.00513; B – 0.000306; Cd – 0.000249; Cr – 0.00097; Cu – 0.000744; Li – 0.037; Ni – 0.000274; Pb – 0.0042; Sr – 0.00116; and Zn – 0.00622.

### 2.4 The photosynthetic performance analyses

The photosynthetic efficiency, as measured by induced chlorophyll fluorescence, was determined according to the protocol described by Krause and Weis [24]. The Maximum quantum yield of photosystem II (F_v_/F_m_), an essential parameter indicating the status of photochemical reactions, was measured in vivo, in situ, using a portable Plant Stress Meter (BioMonitor S.C.I. AB, Sweden), with fifteen sun leaves from around the periphery of the crown of each tree (n = 15). Prior to measurement, leaves were acclimated to darkness for 30 min. The chlorophyll was excited for 2 s by actinic light with a photon flux density of 200 and 400 μmol m^−2^ s^−1^.

### 2.5 Data analysis

The availability of PTE in the soil was estimated using the availability index (AR index) [25], which is calculated as the ratio between the DTPA content of the element (C_DTPA_) and its total content in the soil (C_tot_) and expressed as a percentage:

(1) $$\text{AR}=C_{\text{DTPA}}/C_{\text{tot}}*100$$

To evaluate the potential of the studied plant species for the accumulation of potentially toxic elements (PTEs), the following key indices were used:

Enrichment coefficients for roots (ECR) were calculated by dividing the elemental contents present in plant roots by the total elemental content in the soil [26,27]. A plant is considered to have phytoextraction potential if its ECR value is greater than 1, while plants with ECR values less than 1 are considered excluders [28]. Enrichment coefficients for leaves (ECL) were used to determine the accumulation of PTEs in leaves. This coefficient was derived by dividing the PTE content in plant leaves by the corresponding content in soil [27]. Values close to 1 (e.g., 0.90 or 0.80) indicate the potential suitability of plants for remediation [27,29].

The translocation factor (TLF), represented as the proportion of element content in leaves to roots, was used to estimate the efficiency of PTE uptake and their transfer to leaves [27,30]. Plants with TLF greater than 1 are classified as phytoextractor, and TLF is a critical factor for phytoremediation due to its importance in element uptake and transfer [31].

The Metal Accumulation Index (MAI), based on Liu [32], provides an overall assessment of plant performance in terms of PTE accumulation in leaves:

(2) $$\text{MAI}=\left(1/N\right)\;\sum_{j=1}^{N}Ij$$

where N is the total number of PTEs analyzed and Ij = x/dx is the sub-index for the variable j, which is determined by dividing the mean (x) of each PTE by its standard deviation (dx). Specifically, the MAI for this study was calculated as follows:

(IAs + IB + ICd + ICr + ICu + ILi + INi + IPb + ISr + IZn)/10 = (xAs/dAs + xB/dB + xCd/dCd + xCr/dCr + xCu/dCu + xLi/dLi + xNi/dNi + xPb/dPb + xSr/dSr + xZn/dZn)/10.

The MAI was determined for each sampling site.

The relationships between the contents of the studied elements in soil, roots, and leaves were analyzed using the non-parametric Spearman correlation due to the non-normal distribution. The same was used to determine the relationship between contents of PTEs in leaves, with photosynthetic efficiency parameter (Fv/Fm). Statistically significant differences between element contents in the root and leaf samples were determined using the one-way test ANOVA with post-hoc least significant difference (LSD) test.

Significance levels for ANOVA tests and Spearman correlations were marked with * for p < 0.05, ** for p < 0.01, and *** for p < 0.001.

Descriptive and multivariate statistical analyzes were performed using Statistica 12.0 [33] and OriginPro 2023b [34] software. All attached maps were created with the ArcGis program ArcMap 10.6.1 [35].

## 3 Results and discussion

### 3.1 Physical and chemical properties of the soils

**Fig. 2:**
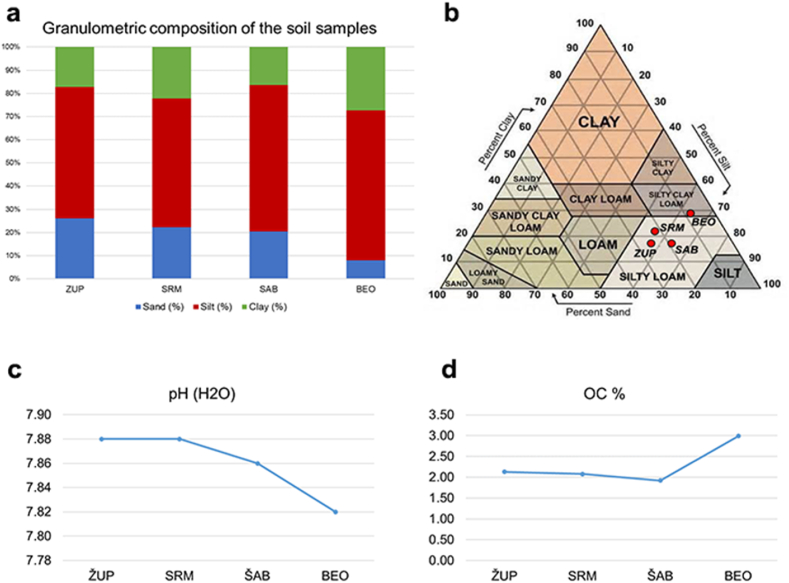
Physical and chemical properties of the studied soils: a) percentage of sand, silt and clay in the soils; b) texture classification of the soils; c) pH of the studied soils; d) percentage of organic matter in the soils.

The distribution of particle sizes in the soil samples studied, the pH, and the percentage of organic carbon (OC) at a depth of 0–30 cm are shown in Fig. 2. The total percentage of sand (particle size 0.06–2.0 mm) ranged from 7.91% in BEO to 26.08% in ZUP. The percent silt (particle size 0.002–0.06 mm) ranged from 55.57% in SRM to 64.75% in BEO. Particles less than 0.002 mm in diameter constituted the clay fraction, whose percentage ranged from 16.36% in SAB to 27.34% in BEO. A study of the total sand content in the mechanical composition of the soil showed a decrease in the lower part, associated with an increase in the proportions of silt and clay (Fig. 2a). The entire lower part of the river course was characterized by a dominance of silt and clay.

With respect to the quantified content of sand, silt and clay in the studied samples, the soil was classified on the basis of its granulometric composition using a texture triangle (Fig. 2b). The surface layers of the soils from ZUP, SRM, and SAB were classified as silty clays. In contrast, the surface layer of the soil collected in BEO had a higher percentage of clay particles and silt, which placed it in the silty clay textural class. These shifts in granulometric composition exert a profound influence on the overall physical and chemical properties of the soil. Soils rich in clay particles tend to bind PTEs and prevent them from being easily removed by precipitation and flooding [36].

The potential for soil contamination with potentially toxic elements depends on their bioavailability. To assess the ecological status of the soil, it is essential to determine the interplay between the physical and chemical properties of the soil and the accessibility of the elements [10,37,38]. Among the various factors, soil pH plays the most important role in determining the bioavailability, mobility, and leaching potential of chemical elements in soil [10,11,37]. In general, the ability of soil to bind most chemical elements increases with higher pH values and usually peaks in the neutral pH range. However, there are some exceptions, such as As, B, and Cr, which become more mobile under alkaline conditions [10]. Conversely, the majority of potentially toxic elements tend to be more available in soils with acidic pH [11]. Conversely, when pH is greater than 7, there is an increased risk of deficiency of essential micronutrients, including B and Zn [39]. Most plants can tolerate soil pH between 5.5 and 6.5 [40]. The pH (H_2_O) values of the studied soils show a relatively small variation, ranging from 7.82 in BEO to 7.88 in ZUP and SRM (Fig. 2c). Given the measured pH values, the studied soils fall into the category of slightly to moderately alkaline soils, as defined by the Soil Science Division Staff [20].

The content of organic carbon (OC %) in the studied soils ranges from 1.92% in SAB to 2.99% in BEO (Fig. 2d). The presence of organic matter is important due to its influence on various soil properties, as it acts as a binder for chemical elements and subsequently reduces their bioavailability [10,11,41]. Organic matter has a strong binding effect on Cr, and Pb, a relatively weak effect on Cd and Ni, and the weakest effect on elements such as Zn [11,42].

In general, the alkaline soil reaction and the relatively low OC content act together to limit the mobility and availability of most of the potentially toxic elements tested [10,37,43].

### 3.2 PTEs in soils

**Fig. 3:**
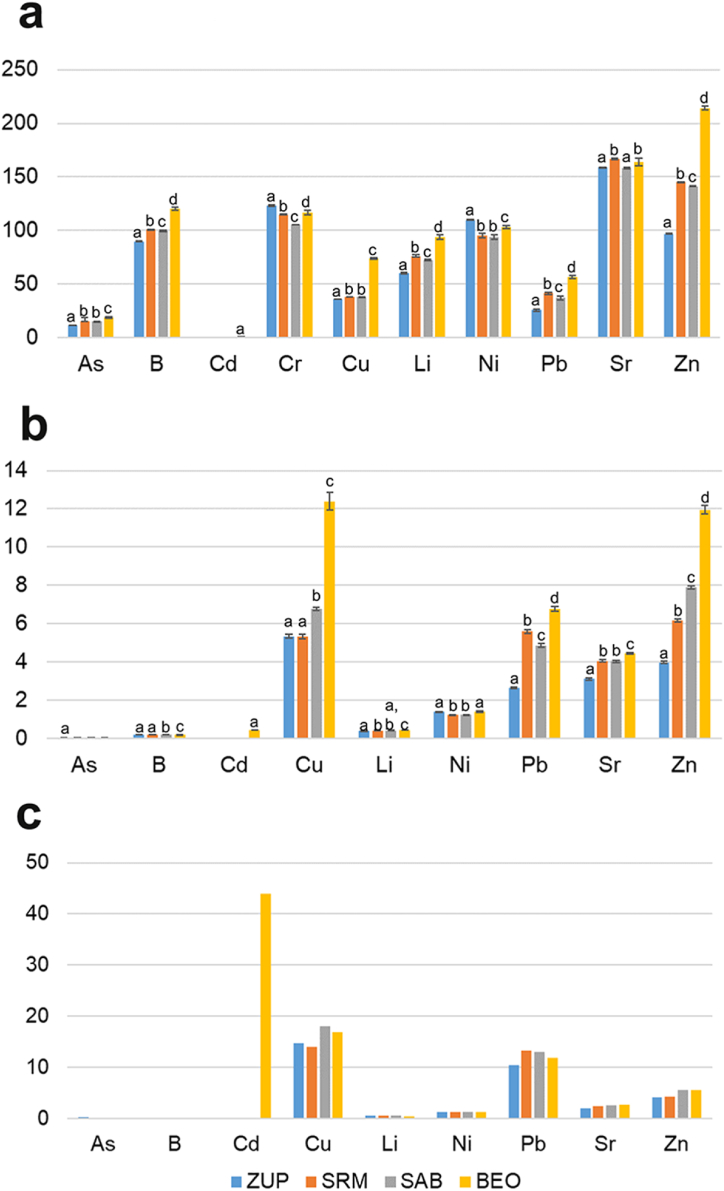
PTE content in soils expressed in mg kg^−1^: a) Total content of PTEs in soils; b) DTPA content of PTEs in soils; c) AR index – availability of PTEs presented in percents (%). Results that do not share a common letter have a statistically significant difference in means according to ANOVA (Tukey LSD post-hoc test).

The total and DTPA content of the chemical elements in the soil samples of the studied sites and the AR availability index are shown in Fig. 3. Of the 10 chemical elements studied in the soil (As, B, Cd, Cr, Cu, Li, Ni, Pb, Sr, and Zn), the total and available Cd content was below the quantification limit of the instrument (<0.000249 mg kg^−1^) at most sites. The presence of DTPA available Cr fractions was also not detected in any of the soil samples studied (<0.00097 mg kg^−1^). By using a DTPA solution, it is possible to evaluate the elemental fractions accessible to plants. When these fractions enter the toxic content range, they have the potential to cause morphological and physiological damage [44,45].

To evaluate the level of contamination of the studied soils, several guidelines were used: the natural background of the study area [5], the natural background for soils in Europe (background value) [46], the average content of these elements in soils in the world [11,43], and the critical range of elements for plants [47]. Details on the content ranges of the PTEs from the above guidelines are presented in Table S2.

When analyzing soil samples from the Sava River, it was found that the maximum levels of As, Cd, Cr, Cu, Ni, Pb, and Zn measured at certain sampling sites were higher than the previously calculated natural background of these elements for the studied area [5]. Compared to the natural background values proposed for European soils [46], higher values were measured for Cr and Ni. All elements studied are above the average value for global soils [11,43]. These results indicate a high anthropogenic pressure, where local soil pollution with certain PTEs may also affect the survival of plants in the coastal zone of the Sava River.

The elements present in the studied area in potentially toxic concentrations for plants [47] are: Cr, whose content in the soil exceeds the critical range for plants of 100 mg kg^−1^, then Cu, which is within the critical range for plants (60–125 mg kg^−1^), and Ni, which also exceeds the critical range for plants of 100 mg kg^−1^ at the ZUP and BEO sites. Similarly, the Zn content is in the critical range for plants (70–400 mg kg^−1^) at all locations (Fig. 3a).

The obtained results of the contents of As, Cd, Cr, Cu, Ni, Sr, and Zn in the soils are similar to previous studies on their content in the Sava River while the content of Pb in this study was lower [5,6,[48], [49], [50]]. In comparison with watercourses on the territory of Europe exposed to the similar anthropogenic pressure, it can be stated that the soils in the lower reaches of the Sava are on average less polluted than the soils in the Danube, Elba, Guadiamar and Leie, with the exception of Ni, which was higher at the studied sampling sites downstream of the Sava [[51], [52], [53], [54]].

The analysis of the DTPA fraction of plant available elements shows that the content of the analyzed PTE was below the critical range for plants at all studied sites (Fig. 3b). Accordingly, the values of the availability coefficient (AR index) for As, B, Li, Ni, Sr and Zn were low (<10%), and for Cu and Pb they were <20%, indicating their low phytotoxicity. (Fig. 3c). The AR index for Cd (>40%) was relatively high, indicating its potential phytotoxicity to plants (Fig. 3c). Still, having in mind that the total content of Cd was very low (0.98 mg kg^−1^), the potential for phytotoxicity of this element is actually low. Compared to previous research on the available PTE contents in riparian soils of the Sava River, the obtained results show much lower values, which is most likely due to differences in the analytical methods [6]. The exception was a similar availability of Cu.

### 3.3 PTEs in *Populus alba*

In this study, reference values were used to analyze and evaluate *Populus alba* for the uptake and accumulation of As, B, Cd, Cr, Cu, Li, Ni, Pb, Sr, and Zn, determining the ranges of deficit, optimum, or toxicity to plants for each of the elements studied [11,37]. To analyze and estimate the potential of the studied species for bioindication and biomonitoring of pollution, the contents of PTE in soil, roots and leaves were corelated. In addition, enrichment coefficients for roots (ECR), leaves (ECL) and translocation factors (TLF) were used to assess and estimate the potential of the studied species for phytoremediation of soils polluted with these elements [30].

In both natural and anthropogenically modified habitats characterized by elevated levels of PTE, plants develop adaptations aimed at tolerating the toxic effects of these elements in soil. These adaptations are based on accumulation (accumulator plants) and exclusion (excluder plants) [8]. The first group of plants has the ability to absorb and accumulate elements in their leaves that exceed their content in the soil and roots (ECL >1, TLF >1) [55], and they are important for phytoremediation processes of polluted soils (phytoextraction). The second group, the exclusion plants, are species that tolerate toxic element levels in the soil by taking up low levels PTE through active exclusion at the root level, i.e. by immobilizing elements in the rhizosphere zone by secreting root exudates and storing the taken-up elements in the cell walls and vacuoles of the root cells. In this group of plants, the root ECR can be greater or less than 1, but the TLF must always be less than 1 [56,57].Pollutant-tolerant plants take up and transport low PTE levels relative to their total soil content (ECR <1, TLF <1) [58,59].

The content of the studied chemical elements in the leaves and roots of *P. alba* is shown in Table 1. The average content of elements in the roots varied in the following order: Zn > Sr > B > Li > Cu > Cr > Pb > Ni > As > Cd, while the average content of elements in the leaves varied in the following order: Zn > B > Sr > Cu > Cd > Pb > Ni > Cr > As=Li.

**Table 1:** Content of elements in roots and leaves of *P. alba*; mean and standard deviation in parentheses; values are given in mg kg^−1^.

PTE content in *P. alba* roots										
Sampling site	As	B	Cd	Cr	Cu	Li	Ni	Pb	Sr	Zn
**ZUP**	2,56^a^ (0,44)	12,72^a^ (0,48)	0,77^a^ (0,00)	**6,82**^a^**(0,53)**	6,42^a^ (0,45)	**13,31**^a^**(0,48)**	9,32^a^ (0,18)	2,97^a^ (0,48)	**34,19**^a^**(0,84)**	28,17^a^ (0,32)
**SRM**	2,14^a^ (0,72)	11,60^b^ (0,40)	0,52^b^ (0,00)	**6,17**^a^**(0,12)**	5,89^a^ (0,61)	**8,19**^**b**^**(1,62)**	5,97^b^ (0,87)	4,92^b^ (0,33)	27,57^b^ (0,18)	29,31^a^ (3,34)
**SAB**	2,52^a^ (0,52)	14,96^c^ (0,02)	2,06^c^ (0,00)	**8,65**^**b**^**(0,22)**	11,68^b^ (0,18)	**14,79**^**c**^**(0,12)**	6,11^b^ (0,43)	11,07^c^ (0,36)	**40,50**^**c**^**(0,22)**	42,35^b^ (0,31)
**BEO**	1,83^a^ (0,36)	7,31^d^ (0,18)	2,06^c^ (0,00)	3,61^c^ (0,63)	4,56^c^ (0,09)	**6,83**^**b**^**(0,36)**	2,56^c^ (0,11)	5,81^b^ (0,89)	26,24^d^ (0,29)	36,95^c^ (0,15)
**PTE content in *P. alba* leaves**										

**ZUP**	<LoQ	47,25^a^ (0,17)	0,77^a^ (0,00)	0,50^a^ (0,11)	4,09^a^ (0,28)	<LoQ	1,40^a^ (0,08)	0,56^a^ (0,23)	**63,36**^a^**(0,75)**	50,64^a^ (2,32)
**SRM**	<LoQ	**107,35**^**b**^**(0,53)**	0,77^a^ (0,00)	0,36^b^ (0,11)	3,67^b^ (0,00)	<LoQ	<LoQ	0,45^a^ (0,14)	**88,27**^**b**^**(0,97)**	84,18^b^ (0,60)
**SAB**	<LoQ	**135,26**^**c**^**(0,24)**	1,03^b^ (0,00)	0,22^c^ (0,00)	7,53^c^ (0,13)	<LoQ	<LoQ	1,63^b^ (0,83)	**69,56**^**c**^**(0,23)**	**238,70**^**c**^**(14,04)**
**BEO**	<LoQ	**106,52**^**b**^**(0,08)**	1,55^c^ (0,00)	0,43^a^^,b^ (0,00)	5,50^d^ (0,00)	<LoQ	0,82^b^ (0,00)	0,71^a^ (0,51)	**67,53**^**d**^**(1,80)**	**175,90**^**d**^**(1,86)**

**Deficit for plants**^a^	–	20–70	–	–	2–5	–	–	–	–	10–20
**Optimum for plants**^a^	1-1,7	10–100	0,002-1	0,1–0,5	5–30	3–5	0,1-5	0,2-10	1–10	27–150
**Toxicity for plants**^a^	5–20	>50	5–30	5–30	20–100	5–50	10–100	30–300	>30	100–400

a Kabata-Pendias 2011; values in the toxic range for plants are denoted in bold; values in the deficit range are shaded; <LoQ – below the limit of quantification; Results that do not share a common letter have a statistically significant difference in means according to ANOVA (Tukey HSD post-hoc test).

The results show that the species *P. alba* accumulates certain elements (B, Cr, Li, Sr, and Zn) in contents considered toxic to plants. The results of measuring the content of elements in roots showed the following: Cr > 5 mg kg^−1^ was measured in ZUP, SRM, and SAB; and Li > 5 mg kg^−1^ in all studied locations. In the roots of *P. alba*, contents above the toxic range for Sr (>30 mg kg^−1^) were measured in ZUP and SAB (Table 1). The obtained results indicate the presence of local point anthropogenic sources for these PTEs. Toxic contents of B, Sr, and Zn were also measured in *P. alba* leaves at specific sites: B (>100 mg kg^−1^) was measured in SRM, SAB, and BEO; Sr (>30 mg kg^−1^) in all sites, while Zn (>100 mg kg^−1^) was measured in SAB and BEO (Table 1).

On the other hand, low contents of essential elements were measured in the roots of *P. alba*, which were in the deficit range for plants, namely B (<10 mg kg^−1^) in all sampling sites and Cu (<5 mg kg^−1^) in BEO, and Cu deficiency was also measured in the leaves at ZUP and SRM (Table 1). The Cu deficiency in the roots and leaves of *P. alba* may be caused by the alkaline reaction of the soil, the low organic matter content, and the low clay content in the soil of the studied sites [10,11,60]. This was confirmed by the correlations between the PTE contents in roots and leaves and the physical and chemical properties of the soils (see Fig. S1in the Supplementary). Typical symptoms of copper deficiency includes, among other symptoms, disruption of photosynthetic activity [61], which was the case in this research (Table 1, Fig. 5).

The results show that this species has a high affinity for the uptake and accumulation of certain PTE from the soil and that this is a characteristic of the species. Comparing the measured contents of Cd, Cu, Ni, Pb, and Zn in the leaves of *P. alba* in the coastal soils at the studied sites on the Sava River with those in other rivers in Europe, it can be concluded that the expected higher contents of Cd and Zn in the leaves of *P. alba* were measured in the riparian zone of the Guadiamar River in Spain, which was affected by the discharge of toxic mine sludge, while the measured contents of Cu, Ni, and Pb were similar [9,62]. Similarly, higher content of Cd and Zn were measured in *P. alba* leaves at polluted sediment dumps along the Scheldt River in Belgium, while levels of Cu and Pb were similar to those measured in samples analyzed in this study [63]. The lower accumulation of Cd and Zn in *P. alba* leaves might be related to the lower content of these elements in the coastal soils of the Sava River, compared to the content of these elements in the soils along the Guadiamar and Scheldt rivers.

Similar content of Cd, Cr, Cu, Pb, and Zn were found in *P. alba* at the studied sites and in the roots and leaves of the *P. alba* clone *'Villafranca'* in uncontaminated habitats near the town of Kórnik and near the tannery waste dump near the town of Kępice (Poland) [64]. An exception is the very high Cr content that the clone *'Villafranca'* accumulated in the roots at the tannery landfill, which indicate the potential for accumulation of this element in the roots. This is confirmed by the measured toxic levels of Cr in the roots of *P. alba* in the coastal zone of the Sava River (Table 1). Also, the study of the content of B, Cd, Cr, Ni, and Zn in the roots and leaves of *P. alba* and the *P. tremula × P. alba* hybrid under experimental conditions showed that *P. alba* has the potential to accumulate extremely high concentrations of these elements [63,[65], [66], [67]]. Elevated levels of B have also been measured in leaves of *P. alba × glandulosa* growing on a wood industry landfill in New Zealand [68].

The results of the measurement of the content of Sr in the leaves and roots, and of Li in the roots of *P. alba* show the accumulation of elevated contents of these elements. On average, Sr concentrations measured in leaves were twice those considered toxic to plants (Table 1). However, the available literature does not provide data on the accumulation of Sr and Li for the species *P. alba*, nor on the potential toxic effects of these elements, and additional studies are needed to more accurately determine the species' potential for Sr and Li accumulation.

**Fig. 4:**
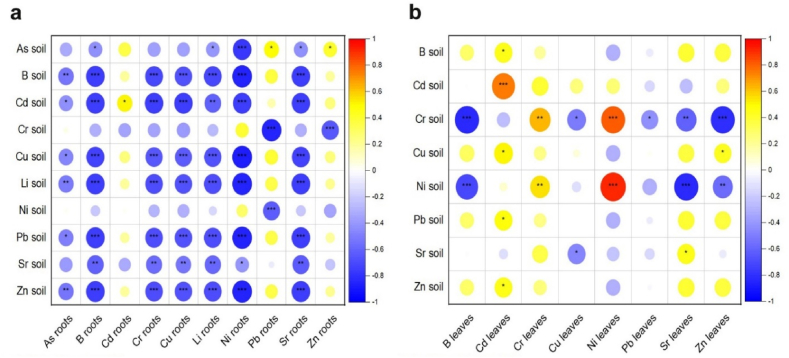
a) correlation of PTEs in roots and soils; b) correlation of PTEs in leaves and soils; Level of statistical significance: *p < 0.05; **p < 0.01; ***p < 0.001.

The results of these and other studies indicate the potential of *P. alba*, as well as several other species of this genus, for biomonitoring pollution of various soil types, including coastal soils of large rivers. As mentioned above, the toxic contents of PTE measured in the plant material, especially in the leaves of *P. alba* from the riparian zone of the Sava River, are the result of the presence of toxic contents of these elements in the soil, which was also confirmed by the results of the correlation analysis (Fig. 4b). Namely, significant positive correlations were found between the content of Cd, Cr and Ni in the soil and in the leaves, which indicates the potential for bioindication and monitoring of pollution of the soils in the riparian zone with these PTEs. The correlations between soil and root content PTE of were mostly statistically significant, but negative (Fig. 4a).

**Fig. 5:**
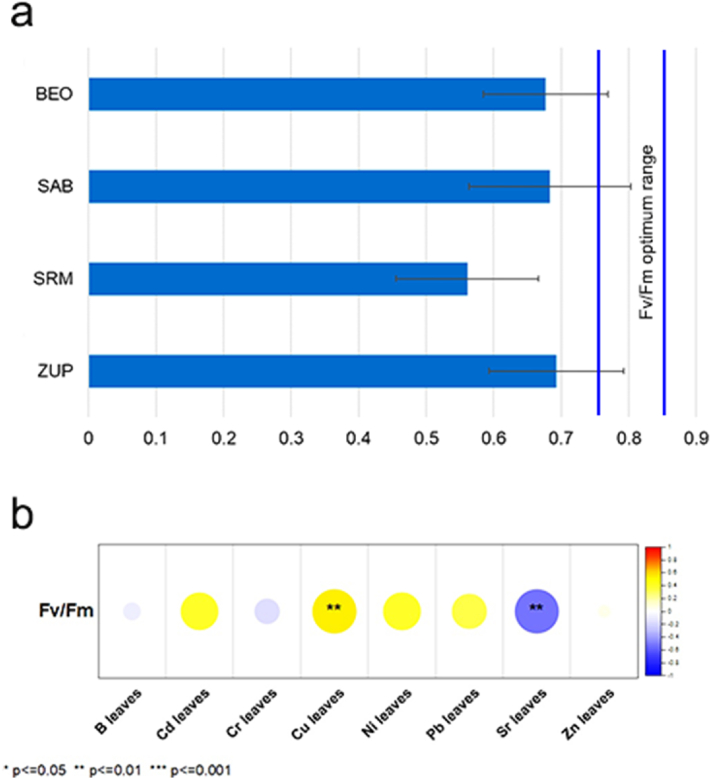
The photosynthetic performance of *P*. *alba*: a) mean Fv/Fm with standard deviation; b) correlations of PTEs in leaves with Fv/Fm.

The results obtained show that the ECR <1 was determined for all elements in the studied sites, except for Cd in BEO (Table 2), while ECL higher than 1 was calculated for B (in SRM and SAB), Cd (in BEO), and Zn (in SAB) (Table 3). On the other hand, the TLF >1 was calculated for B, Sr, and Zn in all studied sites, for Cd in ZUP and SRM, and for Cu in BEO (Table 4), indicating the phytoextraction abilities for these elements, especially B and Zn (both ECL and TLF >1 and measured toxic contents in leaves) (Table 1, Table 3, Table 4). In general, the results of this study confirm the high mobility of B, Sr, and Zn in soils, as well as their transport and accumulation in leaves (Table 2, Table 3, Table 4). The accumulation potential for boron and zinc in the leaves of *P. alba* is supported by earlier studies on this species [9,16], while translocation of Sr in leaves was found in *P. nigra* [7].

**Table 2:** Enrichment coefficients for roots (ECR).

Lokalitet	As	B	Cd	Cr	Cu	Li	Ni	Pb	Sr	Zn
**ZUP**	0,22	0,14	/	0,06	0,18	0,22	0,08	0,12	0,22	0,29
**SRM**	0,14	0,12	/	0,05	0,16	0,11	0,06	0,12	0,17	0,20
**SAB**	0,17	0,15	/	0,08	0,31	0,20	0,06	0,30	0,26	0,30
**BEO**	0,10	0,06	**2,10**	0,03	0,06	0,07	0,02	0,10	0,16	0,17

ECR>1 values are denoted in bold.

**Table 3:** Enrichment coefficients for leaves (ECL).

Lokalitet	As	B	Cd	Cr	Cu	Li	Ni	Pb	Sr	Zn
**ZUP**	/	0,53	/	0,00	0,11	/	0,01	0,02	0,40	0,52
**SRM**	/	**1,07**	/	0,00	0,10	/	/	0,01	0,53	0,58
**SAB**	/	**1,36**	/	0,00	0,20	/	/	0,04	0,44	**1,69**
**BEO**	/	0,88	**1,58**	0,00	0,07	/	0,01	0,01	0,41	0,82

ECL>1 values are denoted in bold.

**Table 4:** Translocation factor (TLF) for studied PTEs and Metal Accumulation Index (MAI).

Lokalitet	As	B	Cd	Cr	Cu	Li	Ni	Pb	Sr	Zn	MAI
**ZUP**	/	**3,71**	**1,00**	0,07	0,64	/	0,15	0,19	**1,80**	**1,80**	**42,33**
**SRM**	/	**9,25**	**1,50**	0,06	0,62	/	/	0,09	**3,20**	**2,87**	**44,03**
**SAB**	/	**9,04**	0,50	0,03	0,64	/	/	0,15	**1,72**	**5,63**	**94,29**
**BEO**	/	**14,57**	0,75	0,12	**1,21**	/	0,32	0,12	**2,57**	**4,76**	**146,50**

TLF>1 values are denoted in bold.

ECR>1 and ECL>1 for Cd in BEO were determined, indicating the potential of *P. alba* for phytoremediation of this element (Table 2, Table 3). At all sites studied, the TLF<1 was determined for Cr, Ni, and Pb, indicating that *P. alba* excludes these PTEs. The translocation factor was not calculated for As and Li because these elements were below the quantification limit of the instrument in the leaves of *P. alba*. Based on the MAI, this species has a very high accumulation potential for PTEs (Table 4). For comparison, Hatami-Manesh et al. [69] found the highest MAI for the accumulation of Cd, Cr, Cu, Ni, Pb, and Zn in *Salix alba* (MAI = 6.98), while Nadgórska–Socha et al. [70] found the highest MAI for the accumulation of Cd, Cu, Fe, Mn, Pb, and Zn in *Betula pendula* (MAI = 27.2). The MAI for Li and Sr (MAI = 60) in *Populus nigra* was calculated in previous study by Miletić et al. [7].

### 3.4 The photosynthetic performance of *Populus alba*

The measurement of the photosynthetic efficiency parameter Fv/Fm is a widely used method to evaluate the photosynthetic efficiency of plants that provides a reliable assessment of the maximum efficiency of the photosystem II (PSII) [71,72]. The Fv/Fm ratio is a valuable indicator of photosynthetic efficiency and is closely related to the number of functional reaction centers of PSII. It plays a critical role as an early and sensitive marker for assessing photoinhibitory damage to the photosynthetic apparatus of plants, especially under stress conditions [18]. Optimal values for the photosynthetic efficiency parameter (Fv/Fm), ranging from 0.750 to 0.850 [73], have been used as a guideline for evaluating photosynthetic performance of *P. alba*. A decrease in Fv/Fm values occurs in response to various stressors and decreased photosynthetic levels [74,75]. PTEs are one of the most important stress factors for photosynthesis [17]. Measured Fv/Fm values, and the correlations between the measured PTEs in the leaves and Fv/Fm are shown in Fig. 5.

The measured values of the photosynthetic efficiency parameter Fv/Fm for *P. alba* (from 0.561 at SRM to 0.693 at ZUP; Fig. 5a) were below the optimal range determined for deciduous trees [73]. Previous studies have shown that poplar clones decreased Fv/Fm values with prolonged waterlogging [76], and he similar Fv/Fm rates were found in *Populus tomentosa* wild type, under oxidative stress [77]. In *Populus alba* growing on thermal power plant fly ash landfills containing abundant PTEs (e.g., As, B, Mo, Cu), a strong reduction in photosynthetic efficiency was observed due to accumulation of high boron concentrations in the leaves [78]. Similar studies at the habitats with excessive concentrations of PTEs in the ash as well as essential elements such as Cu, Mn, and Zn in the plant tissue showed a significant decrease in Fv/Fm in the woody species *T. tetrandra* between 0.377 and 0.666, which corresponds to an overall reduction of 21% compared to the natural/control site [79].

Spearman correlation results showed a statistically significant positive correlation between Fv/Fm and Cu (0.530, p < 0.01) and a negative correlation between Fv/Fm and Sr in leaves (−0.534, p < 0.01) (Fig. 5b). The lower photosynthetic performance of *P. alba* could be the result of a Cu deficit in leaves. A similar effect of reduced copper content on the photosynthetic efficiency parameter was shown by Shahbaz et al. [61], who tested white poplar hybrids (*P. tremula x P. alba*, INRA 717-1B4) under experimental conditions. Although *P. alba* accumulated toxic Sr levels in their leaves (Table 1), this does not appear to have any effect on the reduced photosynthetic performance of this species. This result is consistent with the existing but sparse and occasionally contradictory literature. Namely, Moyen and Roblin [80] and Chen et al. [81] in similar studies, with different plant species, yielded conflicting results suggesting that plant sensitivity to Sr toxicity depends on factors such as Sr concentration, exposure duration, plant age, and species.

Since no visible morphological symptoms were observed on the leaves, it can be assumed that the reduced photosynthetic performance is an adaptation strategy of this species to the elevated and toxic contents of PTEs and oxidative stress caused by the riparian habitat conditions.

## 4 Conclusion

The results of this study have shown phytotoxic contents of Cr, Cu, Ni and Zn in the riparian soils in the lower part of the Sava River. Based on the availability of PTEs, the percent of available Cd was very high, but having in mind its low total content, there is actually a low potential for phytotoxicity of this element. *Populus alba* has accumulated B, Cr, Li, Sr, and Zn in contents that could be considered toxic to plants. Based on the MAI, this species has great potential for the accumulation of investigated elements (As, B, Cd, Cr, Cu, Li, Ni, Pb, Sr, and Zn). Considering the potential for bioindication of pollution of investigated riparian soils with PTEs, *P. alba* has a potential for bioindication of Cd, Cr, and Ni. The accumulation factors (ECR, ECL and TLF) have shown the potential of this species for the phytoextraction of Cd and to a lesser extent for the phytoextraction of B and Zn. Photosynthetic performance was reduced most likely due to Cu deficiency in the leaves, but without visible morphological symptoms. These findings suggest that the reduced photosynthetic performance could be an adaptation strategy of this species to elevated and toxic levels of PTEs. *Populus alba* is one of the predominant species in Europe's riparian forests, mainly found in river valleys. It is therefore a good candidate for phytoremediation and bioindication of soil pollution in riparian areas with PTEs. Considering the contamination of the soils in the lower part of the Sava River with Cr, Cu, Ni, and Zn, we could conclude that this species is suitable for bioindication of Cr and Ni, and remediation of Zn. Further research must be conducted in order to find the species that can be used for bioindication of Cu, Sr and Zn, as well as in the phytoremediation of Cr, Cu, and Ni in riparian soils of the lower course of the Sava River.

## Data availability statement

The data obtained in this study will be available on request.

## CRediT authorship contribution statement

**Zorana Miletić:** Writing – review & editing, Writing – original draft, Conceptualization. **Milica Jonjev:** Methodology, Data curation. **Snežana Jarić:** Validation, Supervision. **Olga Kostić:** Validation, Investigation. **Dimitrije Sekulić:** Software, Investigation. **Miroslava Mitrović:** Writing – review & editing, Writing – original draft. **Pavle Pavlović:** Resources, Funding acquisition.

## Declaration of competing interest

The authors declare that they have no known competing financial interests or personal relationships that could have appeared to influence the work reported in this paper.
