# Supplementary material for: Green solution to riparian pollution: Populus alba L. potential for phytoremediation and bioindication of PTEs along the Sava river
Source: Heliyon. 2024 Mar 18;10(7):e28183. doi: 10.1016/j.heliyon.2024.e28183 (PMC10981065; doi:10.1016/j.heliyon.2024.e28183)
Supplement: Multimedia component 1 [file mmc1.docx]

Green Solution to Riparian Pollution: *Populus alba*'s Potential for Phytoremediation and Bioindication of PTEs along the Sava River

- Supplementary -

Zorana Miletić, Milica Jonjev, Snežana Jarić, Olga Kostić, Dimitrije Sekulić, Miroslava Mitrović, Pavle Pavlović

Department of Ecology, Institute for Biological Research ‘Siniša Stanković’, University of Belgrade, Bulevar despota Stefana 142, Belgrade, Serbia

Corresponding author:

Zorana Miletić,

E-mail: zorana.mataruga@ibiss.bg.ac.rs

ORCID: <https://orcid.org/0000-0003-3353-4810>

Table S.1: Sampling site properties

| **Sampling site** | **Full name** | **State** | **Latitude (°)** | **Longitude (°)** | **Population** | **Climate** | | **Contamination sources** |
| --- | --- | --- | --- | --- | --- | --- | --- | --- |
|  |  |  |  |  |  | **Average temperature** | **Total percipitation** |  |
| **ZUP** | Zupanja | Croatia | 45.075484 | 18.686883 | 12.000 | 11.3°C | 761 mm | Agricultural activities, river navigation, oil, metal and mining industries |
| **SRM** | Sremska Mitrovica | Serbia | 44.913575 | 19.752491 | 38.000 | 11.4°C | 662 mm | Agricultural and urban activities, inland navigation, heavy industry |
| **SAB** | Sabac | Serbia | 44.769900 | 19.699400 | 54.000 | 11.6°C | 699 mm | Agricultural and urban activities, river transport, chemical industry |
| **BEO** | Belgrade | Serbia | 44.768511 | 20.355560 | 1.660.000 | 12.3°C | 663 mm | Urban activities, river transport, thermoelectric plants, untreated urban water, industry |

Table S.2: Guidelines for content ranges of the PTEs used in manuscript; expressed in mg kg^-1^

| **Guideline** | **As** | **B** | **Cd** | **Cr** | **Cu** | **Li** | **Ni** | **Pb** | **Sr** | **Zn** |
| --- | --- | --- | --- | --- | --- | --- | --- | --- | --- | --- |
| **The natural background of the study area**  **(Marković et al. 2018)** | 11,53 | - | 0,68 | 72,52 | 24,12 | - | 41,33 | 44,03 | - | 91,64 |
| **The natural background for soils in Europe**  **(background value; Gawlik and Bidoglio 2006)** | - | - | 1-3 | 50-100 | 50-140 | - | 30-75 | 50-300 | - | 150-300 |
| **The average content of these elements in soils of the world**  **(Kabata-Pendias and Mukherjee 2007; Kabata-Pendias 2011)** | 4,4-8,4 | 10-100 | 0,37-0,45 | 47-51 | 13-23 | 13-28 | 13-26 | 22-28 | 87-210 | 45-60 |
| **The critical range of elements for plants**  **(Alloway 2013)** | 20-50 |  | >2,5 | 75-100 | 60-125 |  | >100 | >100 |  | 70-400 |

Fig. S.1: Correlation of PTEs in leaves and roots with physical and chemical properties of the soils:


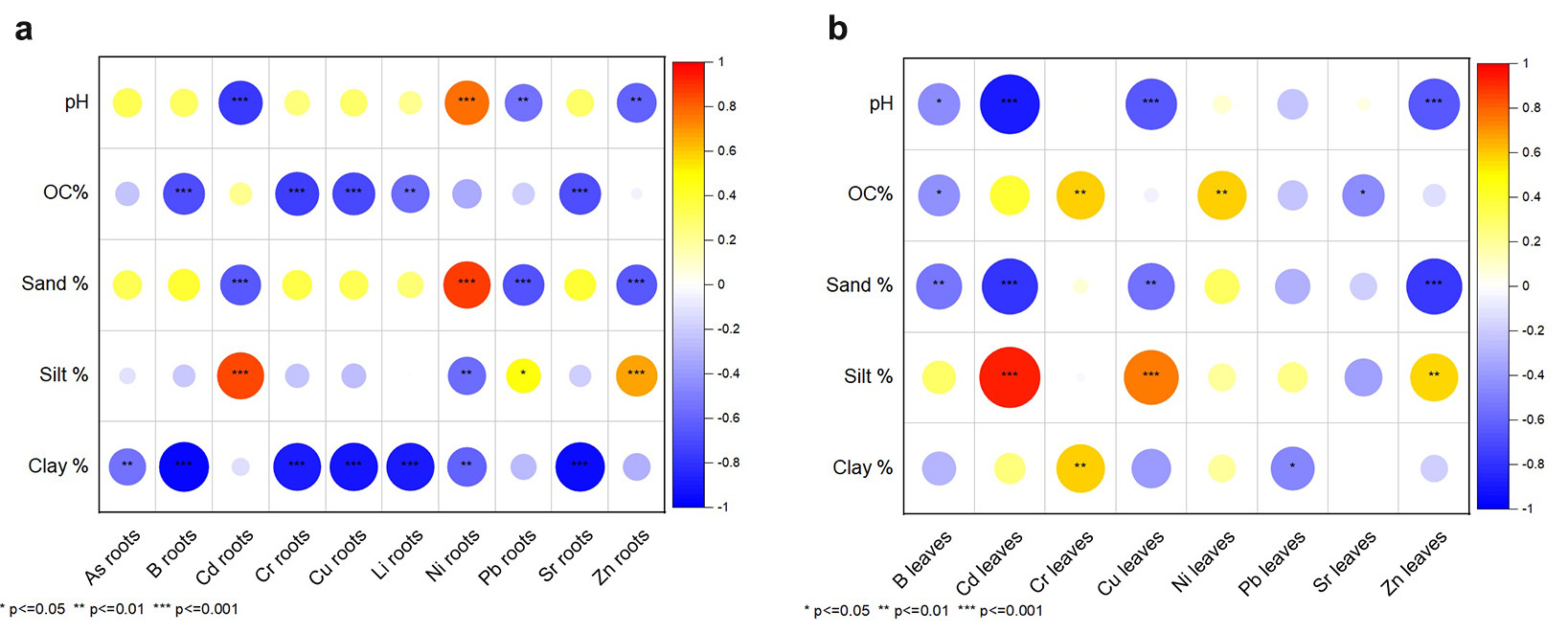


1. correlation of the PTEs in roots with the physical and chemical properties of the soils; b) correlation of the PTEs in leaves with the physical and chemical properties of the soils
